# The Impact of Hybridization on the Volatile and Sensorial Profile of *Ocimum basilicum* L.

**DOI:** 10.1155/2014/824594

**Published:** 2014-01-12

**Authors:** Andréa Santos da Costa, Maria de Fátima Arrigoni-Blank, Maria Aparecida Azevedo Pereira da Silva, Mércia Freitas Alves, Darlisson de Alexandria Santos, Péricles Barreto Alves, Arie Fitzgerald Blank

**Affiliations:** ^1^Department of Agronomic Engineering, Federal University of Sergipe, Avenue Marechal Rondon s/n, 49100-000 São Cristóvão, SE, Brazil; ^2^Department of Food Technology, Federal University of Sergipe, Avenue Marechal Rondon s/n, 49100-000 São Cristóvão, SE, Brazil; ^3^Department of Chemistry, Federal University of Sergipe, Avenue Marechal Rondon s/n, 49100-000 São Cristóvão, SE, Brazil

## Abstract

The aim of the present study was to investigate the volatile and sensorial profile of basil (*Ocimum basilicum* L.) by quantitative descriptive analysis (QDA) of the essential oil of three hybrids (“Cinnamon” × “Maria Bonita,” “Sweet Dani” × “Cinnamon,” and “Sweet Dani” × “Maria Bonita”). Twelve descriptive terms were developed by a selected panel that also generated the definition of each term and the reference samples. The data were subjected to ANOVA, Tukey's test, and principal component analysis. The hybrid “Cinnamon” × “Maria Bonita” exhibited a stronger global aroma that was less citric than the other samples. Hybridization favored the generation of novel compounds in the essential oil of the hybrid “Sweet Dani” × “Maria Bonita,” such as canfora and (E)-caryophyllene; (E)-caryophyllene also was a novel compound in the hybrid “Sweet Dani” × “Cinnamon”; this compound was not present in the essential oils of the parents.

## 1. Introduction

Species of the genus *Ocimum* are widely used and appreciated due to their essential oil that includes several components of interest. *O. basilicum* has the largest market demand among the species and chemotypes and is cultivated for commercial purposes in several countries, such as India, France, Morocco, and Italy [1].

Basil (*Ocimum basilicum* L.) is an herbaceous aromatic plant that can be grown as an annual or perennial according to the region where the plant is cultivated. The stalks grow up to 1 m tall and have greatly branched stems with quadrangular or pubescent branches. The leaves are 1 to 4 cm long and 1.85 to 9.25 cm wide [2]. The leaves are petiolate, opposite, ovate, or toothed and either green or purple. As a medicinal plant, basil is used to treat respiratory disorders, bacterial infections, and bowel parasites; basil also improves digestion [3]. The quality of basil plants is defined by the composition of their essential oil [4].

The essential oil can be extracted from fresh or dry leaves and flowers by three methods: hydrodistillation, steam distillation, and solvent extraction [5]. Basil is used in the food, pharmaceutical, and cosmetic industry [6] as well as for the control of pests [7–9] and plant diseases [10]. A study conducted by [11] using hydrodistillation and steam distillation found similar concentrations of chemical compounds in the essential oils, whereas the amount of essential oil extracted was larger when steam distillation was used.

Basil is classified according to its aroma into categories such as sweet, lemon, cinnamate or cinnamon, camphor, anise, and clove. Variations in the chemical composition of the essential oils are used to differentiate the basil as the following types: European, French, or Sweet; Egyptian, Reunion, or Comoro; Bulgarian, Java, or Methyl Cinnamate; and Eugenol. The European type of basil contains mostly linalool and methyl chavicol [12, 13]. The Cinnamon type of basil is rich in methyl cinnamate [14], the Sweet Dani cultivar in citral (68%) [15], and the Maria Bonita cultivar in linalool (78.12%) [16]. Linalool has been widely studied as an acaricide [17], bactericide, and fungicide [18], and this compound has been successfully used in medicine as a sedative [19, 20] and anticonvulsant [21]. Studies conducted with the Maria Bonita cultivar indicate antinociceptive activity of the essential oil [22], whereas potential anti-Giardia activity has already been shown [23].

The chemical composition of the essential oil of *O. basilicum* was the focus of many studies, and several chemotypes have been reported. Oils rich in 1.8-cineole (22%), linalool (49.7%), methyl chavicol (47%), and methyl cinnamate (65.5%) were found in Brazilian plants [24]. In plants cultivated in the Mongolian desert, the main components were 1.8-cineole (8.54%), linalool (27.26%), (Z)-*α*-bergamotene (10.00%), and methyl chavicol (19.77%) [1]. In 18 basil accessions, linalool and methyl-chavicol were the main components [9]. In southern India, the main components were (E)-methyl cinnamate (34.49%), linalool (28.44%), camphor (13.08%), (Z)-methyl cinnamate (6.90%), and geraniol (3.84%) [25]. Another study found the main components to be 1,8-cineole (7.23%), linalool (66.4%), and (Z)-*α*-bergamotene (7.96%) [26]. In a study conducted with seven species of the genus *Ocimum*, linalool (39.8%), estragol (20.5%), methyl cinnamate (12.9%), eugenol (9.1%), and 1,8-cineole (2.9%) predominated [27]. Among 12 varieties of *Ocimum*, ten exhibited a high percentage of methyl cinnamate (35–80%), one single variety contained caryophyllene, and another variety contained linalool [28].

The production of hybrids by crossing cultivars contributes to the creation of new essential oils for the world market. The most important decision for the hybridization program is the choice of parents [29]; this decision depends on the characteristics to be improved, the type of inheritance of the characteristics, and the available source of germplasm [30]. Genetic variability, adaptability, and evolution of the species are important for the success of any improvement program. An improvement program was shown by [2] in which 55 accessions of *Ocimum* sp. were investigated. These authors observed genotypic variation in the content and yield of essential oil and were able to identify promising genotypes for improvement programs. Recently, [31] showed that basil variability might be improved with hybrid combinations. Studies of such combinations and their effects facilitate the selection of genotypes for genetic improvement programs and the development of cultivars.

The aim of the present study was to assess the impact of hybridization on the volatile and sensorial profile of *Ocimum basilicum* L.

## 2. Materials and Methods

### 2.1. Plant Material and Extraction of Essential Oil

Hybridization was performed in a greenhouse covered with white screen at the Federal University of Sergipe. The cultivars used to perform the crosses were “Cinnamon” (*O. basilicum*), “Sweet Dani” (*Ocimum* × *citriodorum*), and “Maria Bonita” (*O. basilicum*) [31]. To realize hybridization, inflorescences of each plant were selected to serve as pollen recipients (female) and marked with wool yarn; different color was used for the male parent and pollen donor. Every morning (07:00–09:00 am), the crosses were performed using collected inflorescences. The picked flowers (containing pollen) were touched against the stigmas of emasculated flowers. After hand pollination, the inflorescences functioning as female organs were protected with paper bags to prevent the flowers from self-pollinating.

The leaves of three hybrids (“Cinnamon” × “Maria Bonita,” “Sweet Dani” × “Cinnamon,” “Sweet Dani” × “Maria Bonita”) and three cultivars (“Cinnamon,” “Sweet Dani,” and “Maria Bonita”) were used in this work.

The assay was conducted in field of the Research Farm “Campus Rural da UFS” using a randomized block design in triplicate. Each plot consisted of a row of five plants. The spacing between rows was 0.50 m and between plants was 0.50 m. The mean minimum and maximum temperature were 24 and 30°C, respectively.

After three months of field cultivation, when the plants were in full bloom, the aerial parts were harvested and the leaves of the plants were dried in an oven dryer with air renewal and circulation (Marconi model MA-037/5) at 40°C for five days [4]. The extraction of the essential oils from the leaves was performed using a Clevenger-type apparatus [5] for approximately 160 minutes [4].

### 2.2. Identification of the Volatile Compounds in the Essential Oils

The analysis of the essential oil chemical composition was performed in a gas chromatograph coupled to a mass spectrometer (GC-MS) (Shimadzu, model QP 5050A) equipped with an AOC-20i auto injector (Shimadzu) and a fused-silica capillary column (5%-phenyl-95%-dimethylpolysiloxane, 30 m × 0.25 mm id., 0.25 *μ*m film, J&W Scientific). Helium was used as the carrier gas at a flow rate of 1.2 mL/min. The temperature program was as follows: 50°C for 1.5 min, temperature increase at 4°C/min until reaching 200°C, temperature increase at 15°C/min until reaching 250°C and 250°C for 5 min. The injector temperature was 250°C, and the detector (or interface) temperature was 280°C. The injection volume of ethyl acetate was 0.5 *μ*L, the partition rate of the injected volume was 1 : 87, and the column pressure was 64.20 kPa. The mass spectrometer conditions were as follows: ionic capture detector impact energy of 70 eV, scanning speed 0.85 scan/s from 40 to 550 Da.

Quantitative analysis of the chemical constituents was performed by flame ionization gas chromatography (FID), using a Shimadzu GC-17A (Shimadzu Corporation, Kyoto, Japan) instrument, under the following operational conditions: capillary ZB-5MS column (5% phenyl-arylene-95%-dimethylpolysiloxane) fused silica capillary column (30 m × 0.25 mm i.d. × 0.25 *μ*m film thickness) from Phenomenex (Torrance, CA, USA), under same conditions as reported for the GC-MS. Quantification of each constituent was estimated by area normalization (%). Compound concentrations were calculated from the GC peak areas and they were arranged in order of GC elution.

The essential oil components were identified by comparing their mass spectra with the available spectra in the equipment database (NIST05, NIST21, and WILEY8). These libraries allowed for the comparison of spectral data and had a similarity index of 80%. Additionally, the measured retention indices were compared with those in the literature [32]. The relative retention indices (RRI) were determined using the [33] equation and a homologous series of *n*-alkanes (C_8_–C_18_) injected under the chromatography conditions described above.

For each volatile compound, the chromatographic peak area count was calculated, and the value was used as an estimate of the concentration of the given compound in the sample. The data were statistically analyzed by means of principal component analysis (PCA) using the software SAS (*Statistical Analysis System*—*SAS *Institute Inc., North Carolina, USA, 2010).

### 2.3. Sensorial Profile

The aroma profile of each essential oil sample was established using quantitative descriptive analysis (QDA), as described by [34].

First, 10 volunteers were recruited among students and collaborators at the Federal University of Sergipe and selected according to their sensitivity to the basic aromas, olfactory memory, and discriminating power among aromas. Next, using the network method [35] and other procedures described by [34, 36], we developed (i) descriptive vocabulary for the essential oil samples, (ii) a consensual definition of each descriptor, (iii) references for the panel training, and (iv) a form for the descriptive assessment of the aromas perceived in the essential oils (Table 1). The descriptive form, defined terms, and references are depicted in Tables 1 and 2. These tools were used to train the panel in sensory assessment of essential oils.

After the training period was over, the trained panel was subjected to a selection test. Each examiner was requested to assess three samples using the abovementioned descriptive form. The experiment was performed in a fully randomized block design in triplicate. According to the methods suggested by [37], the data generated by each examiner were analyzed by ANOVA (variation sources: samples and repetition) and Tukey's test. To compose the final panel, the examiners who exhibited good discriminating power (*P*F_sample_ ≤ 0.05), good repeatability (*P*F_repetition_ ≥ 0.05), and consensus with the remainder of the panel were selected.

Next, the selected panel assessed all of the essential oil samples in triplicate using the descriptive assessment form (Table 1). The samples were identified by a three-digit code, and the panel members were requested to assess the intensity of each descriptor listed in the descriptive form on a nine-cm scale with “none” on the left end, “strong” on the right end, and “moderate” on the midpoint (Table 1). To avoid sensory fatigue, only three essential oil samples were assessed at each session. The order of the presentation of the samples was balanced among the panel members according to the recommendations of [34, 36].

The data generated by the panel were analyzed by ANOVA (variation sources: sampler, sample and interaction of sampler versus sample) and Tukey's test using SAS software (*Statistical Analysis System*—*SAS *Institute Inc., North Carolina, USA, 2010).

## 3. Results and Discussion

The volatile compounds identified in each essential oil sample, their corresponding linear retention indices, and the relative area expressed as percentage of the total area under the chromatogram can be observed in Table 2. All of the volatile compounds that exhibited a relative area that is equal to or higher than 0.25% in weight in at least one sample also were included in Table 2. The sum of all of the compounds was over 90% (Table 2), showing that they represent the main components of each sample.

Linalool was the main volatile compound of the essential oils extracted from the leaves of the basil genotype “Maria Bonita” and the hybrid “Sweet Dani” × “Maria Bonita” (Table 2). The essential oil profile of the genotype “Maria Bonita” exhibits a rather low number of components, and linalool corresponds to approximately 70% of the total area under the chromatogram (Table 2).

Table 3 shows the description of the olfactory quality of each volatile component listed in Table 2 and the chromatographic peak area count, which allows for the estimation of the concentration of each volatile compound in each sample and the calculation of significant differences among the essential oil samples in their chemical components. The investigated genotypes exhibited significant differences (*P* ≤ 0.05) in the concentration of each volatile compound in the investigated samples (Table 3). These findings suggest that the hybridization of these genotypes induced the generation of compounds in the hybrids that were not present in the parents and an increase or decrease in some compounds present in the parents. The similarities and differences among the essential oils in the volatile quantitative profile are depicted in Table 1.

**Figure 1:**
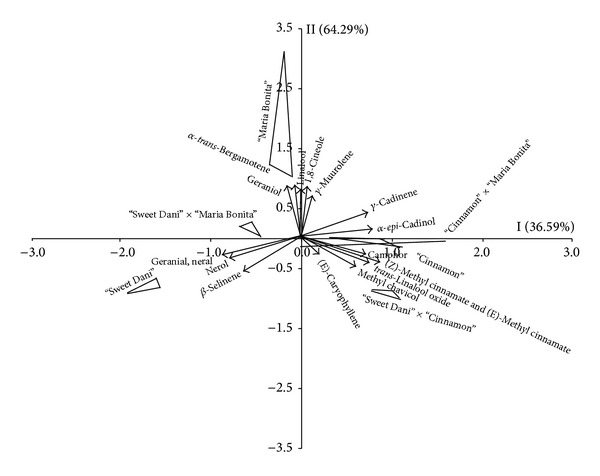
Projection of the sensory descriptors and essential oil samples on the first two principal components.

Figure 1 depicts axes I and II generated by principal component analysis (PCA) of the count values associated with the chromatographic peak areas of each volatile compound. The first two axes explained 56.46% of the variation found among the samples in their respective concentration of volatile compounds. The samples are represented by triangles, where each vertex represents one analytical repetition, and the assessed parameters (volatile compounds) are represented by vectors (Figure 1). To interpret the graphic generated by PCA (Figure 1), the vectors must first be decomposed along axes I and II, and the “weight” of their components on each axis must be analyzed. The volatile compounds 1,8-cineole, gamma-cadinene, alpha-epi-cadinol, (Z)-methyl cinnamate, (E)-methyl cinnamate, camphor, gamma muurolene, and linalool trans-oxide decomposed mostly on axis I (Figure 1). These data suggest that the samples located on the positive side of axis I were unique in composition compared to the other samples. In this case, the essential oil of the hybrid “Cinnamon” × “Maria Bonita” had a greater concentration of volatile compounds than “Sweet Dani” × “Maria Bonita”. The hybrid “Cinnamon” × “Maria Bonita” exhibited a greater concentration of all of the compounds mentioned above except for (E)-methyl cinnamate and gamma-muurolene (Table 3).

The parents did not exhibit the abovementioned compounds in concentrations as high as those found in the oil of hybrid “Cinnamon” × “Maria Bonita”, which suggests the occurrence of heterosis in the hybrid compared to the parents. Therefore, hybridization induced in the hybrid “Cinnamon” × “Maria Bonita” favored the generation of compounds such as linalool trans-oxide, (E)-cinnamate and (Z)-methyl cinnamate that were not present in the essential oils of the parents.

In the hybrids “Sweet Dani” × “Cinnamon” and “Sweet Dani” × “Maria Bonita,” we noted that linalool trans-oxide was the single volatile compound present in the hybrids that was not present in the parents (Figure 1 and Table 3).

The abovementioned instances of heterosis suggest several hypotheses. First, the presence of codominance is suggested. For instance, the female parent exhibits alleles A_1_A_1_, and the male parent exhibits alleles A_2_A_2_ of the same gene, whereas alleles A_1_ and A_2_ by themselves are unable to induce the formation of a given compound. When hybridization occurs, both alleles together might code for the enzyme associated with the synthesis of a novel compound [38]. The structure of some enzymes requires two or more polypeptide chains; thus, allele A_1_ might code for one polypeptide chain, which is not sufficient by itself to form the enzyme, and allele A_2_ might code for another polypeptide chain. Conversely, the presence of both alleles allows for the synthesis of two chains and the consequent formation of the enzyme. A second hypothesis is related to the presence of genetic epistasis or interaction, whereby complementary genetic action or a limiting reaction might occur [39]. In addition, two different enzymes might be needed for the production of the novel compound, whereas each enzyme is coded for by a different gene.

The essential oil extracted from the hybrid “Sweet Dani” and the hybrid “Sweet Dani” × “Maria Bonita” had higher concentrations of nerol, neral, and geranial (Figure 1), which is confirmed by Table 3. Indeed, previous studies [15] showed that geranial was the main compound in the hybrid “Sweet Dani.” Consequently, the volatile profile of the hybrid “Sweet Dani” predominated in its hybridization with the cultivar “Maria Bonita.”

The cultivar “Maria Bonita” had higher geraniol and linalool concentrations (Figure 1 and Table 3). The cultivar “Maria Bonita” is distant from the other samples and remarkably from its hybrids “Sweet Dani” × “Maria Bonita” and “Cinnamon” × “Maria Bonita”, which suggests that its volatile profile differs significantly from the profile of other samples (Figure 1). This finding indicates that upon hybridization, the cultivar “Maria Bonita” exerted less influence than the cultivars “Sweet Dani” and “Cinnamon” on the volatile profile of the hybrids.

The cultivar “Cinnamon” and the hybrid “Sweet Dani” × “Cinnamon” are located at the center of Figure 1, which indicates that these samples not only exhibit similar volatile profiles but also intermediate concentrations of all of the analyzed compounds (Table 3).

Upon describing the essential oils extracted from the hybrids, the trained panel identified aromatic notes such as basil, lemon, orange, lemongrass, lavender, lemon balm, rosemary, cinnamon stick, wood, sweet, and pungent. Most of the descriptors are consistent with the aroma of the volatile compounds identified in the samples (Table 3).

We noted that the sample “Cinnamon” × “Maria Bonita” exhibited a global aroma intensity that is significantly stronger (*P* ≤ 0.05) than that of the other samples because the sample most likely exhibited a greater concentration of most of the volatile compounds present in the hybrids (Table 4).

All of the samples exhibited moderate notes of basil and citric aromas and did not differ in these attributes (Table 4). However, the samples exhibited significant differences (*P* ≤ 0.05) in the intensity of the citric aroma, which was less intense in the hybrid “Cinnamon” × “Maria Bonita.” One reason for this lessened intensity is that the hybrid exhibited a high concentration of several noncitric aromatic volatile compounds, such as 1,8-cineole, which the literature describes as having an aroma of peppermint (http://www.flavornet.org/flavornet.html), gamma cadinene (wood) (http://www.flavornet.org/flavornet.html), and camphor, among others. The aroma of these volatile compounds most likely masked the citric aroma of the linalool present in the hybrid.

The low intensity of the citric aroma exhibited by the hybrid “Cinnamon” × “Maria Bonita” might also be explained by the lack of neral and geranial, which have a citric aroma, in its composition (Table 4).

## 4. Conclusions

Hybridization favored the generation of novel compounds in the essential oil of the hybrid “Sweet Dani” × “Maria Bonita,” such as canfora and (E)-caryophyllene; (E)-caryophyllene also was a novel compound in the hybrid “Sweet Dani” × “Cinnamon”; these compounds were not present in the essential oils of the parents. With hybridization, also new aromas are produced, which were identified by sensory analysis. The hybrid “Cinnamon” × “Maria Bonita” exhibited a stronger global aroma intensity that was less citric than the other samples.

## Figures and Tables

**Table 1:** List of the definitions of the descriptive terms and their corresponding references.

Attribute	Definition	References
Basil	Natural aroma of fresh basil	Sliced fresh basil leaves

Citric	Aroma characteristic of lemon, orange peel, fresh *Cymbopogon densiflorus*, or *Cymbopogon winterianus *	Fresh grated lemon and orange peel and fresh sliced leaves of *Cymbopogon densiflorus* and *Cymbopogon winterianus *

Sweet	Aroma associated with caramel, honey, or sugar	Honey, sugar

**Table 2:** Chemical composition of the *Ocimum basilicum* L. genotypes (mean ± standard deviation).

Peak	RRI	RRI lit	Compound	% peak area					
				“Sweet Dani” × “Maria Bonita”	“Sweet Dani” ×“Cinnamon”	“Cinnamon” × “Maria Bonita”	“Cinnamon”	“Sweet Dani”	“Maria Bonita”
1	1031	1026	1,8-Cineole	3.93 ± 0.40	4.49 ± 0.57	5.73 ± 0.54	4.38 ± 0.77	0.00 ± 0.00	5.35 ± 0.01
2	1086	1084	*trans*-Linalool oxide	0.00 ± 0.00	0.30 ± 0.09	0.14 ± 0.05	0.31 ± 0.10	0.00 ± 0.00	0.00 ± 0.00
3	1100	1095	Linalool	55.63 ± 2.61	15.75 ± 1.32	35.33 ± 3.24	30.78 ± 0.35	0.00 ± 0.00	75.22 ± 1.05
4	1146	1141	Camphor	0.26 ± 0.15	0.50 ± 0.02	0.47 ± 0.05	0.55 ± 0.06	0.00 ± 0.00	0.00 ± 0.00
5	1197	1195	Methyl chavicol	0.00 ± 0.00	3.01 ± 0.31	1.30 ± 0.03	1.03 ± 0.13	0.47 ± 0.41	0.00 ± 0.00
6	1222	1227	Nerol	0.39 ± 0.05	0.00 ± 0.00	0.00 ± 0.00	0.00 ± 0.00	3.13 ± 1.68	0.00 ± 0.00
7	1236	1235	Neral	14.57 ± 1.29	0.57 ± 0.12	0.00 ± 0.00	0.00 ± 0.00	35.68 ± 1.30	0.00 ± 0.00
8	1249	1249	Geraniol	0.82 ± 0.08	0.00 ± 0.00	0.30 ± 0.09	0.00 ± 0.00	0.89 ± 0.23	14.66 ± 0.94
9	1266	1264	Geranial	19.35 ± 1.57	0.84 ± 0.14	0.00 ± 0.00	0.00 ± 0.00	46.16 ± 2.51	0.00 ± 0.00
10	1302	1299	(Z)-Methyl cinnamate	0.00 ± 0.00	6.41 ± 0.49	7.44 ± 0.55	5.75 ± 0.98	0.00 ± 0.00	0.00 ± 0.00
11	1383	1376	(E)-Methyl cinnamate	0.00 ± 0.00	60.26 ± 0.96	43.72 ± 3.36	48.64 ± 1.72	0.00 ± 0.00	0.00 ± 0.00
12	1419	1417	(E)-Caryophyllene	0.49 ± 0.04	0.72 ± 0.08	0.00 ± 0.00	0.00 ± 0.00	1.65 ± 0.29	0.00 ± 0.00
13	1432	1432	*α*-*trans*-Bergamotene	1.15 ± 0.06	0.27 ± 0.04	1.67 ± 0.10	0.00 ± 0.00	0.79 ± 0.08	1.80 ± 0.27
14	1480	1478	*ϒ*-Muurolene	0.39 ± 0.03	0.92 ± 0.10	0.31 ± 0.03	1.15 ± 0.18	0.51 ± 0.24	0.32 ± 0.02
15	1488	1489	*β*-Selinene	0.17 ± 0.01	0.53 ± 0.01	0.00 ± 0.00	0.32 ± 0.12	1.49 ± 0.06	0.00 ± 0.00
16	1511	1513	*ϒ*-Cadinene	0.29 ± 0.01	0.28 ± 0.02	0.84 ± 0.02	1.17 ± 0.09	0.00 ± 0.00	0.49 ± 0.01
17	1641	1638	*α*-*epi*-Cadinol	0.41 ± 0.10	0.73 ± 0.30	0.99 ± 0.22	2.90 ± 0.68	0.00 ± 0.00	0.58 ± 0.15

Total (%)				97.84	95.57	97.94	96.98	90.77	98.43

RRI: relative retention index.

**Table 3:** Chemical composition and aroma of *Ocimum basilicum* L. genotypes.

Compound	Aroma*	Peak area count × 10^4^					
		“Sweet Dani” × “Maria Bonita”	“Sweet Dani” × “Cinnamon”	“Cinnamon” × “Maria Bonita”	“Cinnamon”	“Sweet Dani”	“Maria Bonita”
1,8-Cineole	Mint, sweet	30.43^a^	26.65^a^	25.11^a^	14.99^a^	0.00^a^	32.94^a^
*trans*-Linalool oxide	Sweet, floral, green	0.00^c^	1.71^a^	0.67^bc^	1.08^ab^	0.00^c^	0.00^c^
Linalool	Flower, lavender	431.99^a^	90.93^b^	146.03^b^	101.58^b^	0.00^b^	461.74^a^
Camphor	Camphor	1.82^a^	1.82^a^	2.01^a^	1.83^a^	0.00^a^	0.00^a^
Methyl chavicol	Fragrant, sweet, cooling, fresh, minty	0.00^b^	17.82^a^	5.49^b^	3.45^b^	2.24^b^	0.00^b^
Nerol	Sweet	3.05^b^	0.00^b^	0.00^b^	0.00^b^	15.32^a^	0.00^b^
Neral	Lemon	115.16^b^	3.21^c^	0.00^c^	0.00^c^	171.12^a^	0.00^c^
Geraniol	Rose, geranium	6.37^b^	0.00^b^	1.81^b^	0.00^b^	4.29^b^	90.96^a^
Geranial	Lemon, mint	152.93^b^	4.76^c^	0.00^c^	0.00^c^	221.31^a^	0.00^c^
(Z)-Methyl cinnamate	—	0.00^b^	38.03^a^	31.74^a^	19.70^ab^	0.00^b^	0.00^b^
(E)-Methyl cinnamate	—	0.00^b^	353.88^a^	171.26^ab^	159.71^ab^	0.00^b^	0.00^b^
(E)-Caryophyllene	—	3.84^a^	4.12^a^	0.00^b^	0.00^b^	0.00^b^	0.00^b^
*α*-*trans*-Bergamotene	Fragrant, sweet, fresh	8.89^a^	1.55^a^	7.16^a^	0.00^a^	3.77^a^	37.69^a^
*ϒ*-Muurolene	Herb, wood, spice	3.09^a^	5.31^a^	1.31^a^	3.72^a^	2.41^a^	7.87^a^
*β*-Selinene	Herb	1.29^c^	3.08^b^	0.00^d^	0.97^c^	7.11^a^	0.00^d^
*ϒ*-Cadinene	Wood	2.31^ab^	1.62^ab^	3.54^a^	3.82^a^	0.00^b^	3.03^ab^
*α*-*epi*-Cadinol	Fragrant	3.38^b^	4.00^ab^	4.55^ab^	9.12^a^	0.00^b^	3.70^b^

Means followed by the same lowercase letters in a row do not differ according to Tukey's test at *P* ≤ 0.05.

*Aroma according to [38].

**Table 4:** Average intensity of the outstanding aromatic notes in the hybrids of *Ocimum basilicum*.

Samples	Aromatic notes^1,2^			
	Basil	Sweet	Citric	Global aroma intensity
“Sweet Dani” × “Maria Bonita”	5.75^a^	4.44^a^	4.27^ab^	1.35^c^
“Sweet Dani” × “Cinnamon”	5.35^a^	4.83^a^	4.92^a^	3.86^b^
“Cinnamon” × “Maria Bonita”	5.68^a^	4.80^a^	2.97^b^	7.74^a^

^1^Means followed by the same letters in a column do not differ at *P* ≤ 0.05.

^2^0: weak; 9: strong.
